# Effect of *Hibiscus sabdariffa* extract on high fat diet–induced obesity and liver damage in hamsters

**DOI:** 10.3402/fnr.v59.29018

**Published:** 2015-10-15

**Authors:** To-Wei Huang, Chia-Ling Chang, Erl-Shyh Kao, Jenq-Horng Lin

**Affiliations:** 1 Department of Life Sciences, National Chung Hsing University, Taichung, Taiwan; 2 Department of Nursing, China Medical University Hospital, Taichung, Taiwan; 3 Department of Beauty Science and Graduate Institute of Beauty Science Technology, Chienkuo Technology University, Changhua, Taiwan

**Keywords:** *Hibiscus sabdariffa*, obesity, adipogenesis

## Abstract

**Background:**

Obesity is a chronic metabolic disorder associated with an increase in adipogenesis and often accompanied with fatty liver disease.

**Objective:**

In this study, we investigated the anti-obesity effects of *Hibiscus sabdariffa* water extract (HSE) *in vivo*.

**Method:**

Eight-weeks-old male mice were divided into six groups (*n*=8 per group) and were fed either normal feed, a high fat diet (HFD), HFD supplemented with different concentrations of HSE, or HFD supplemented with anthocyanin. After 10 weeks of feeding, all the blood and livers were collected for further analysis.

**Results:**

Mesocricetus auratus hamster fed with a high-fat diet developed symptoms of obesity, as determined from their body weight change and from their plasma lipid levels. Meanwhile, HSE treatment reduced fat accumulation in the livers of hamsters fed with HFD in a concentration-dependent manner. Administration of HSE reduced the levels of liver cholesterol and triglycerides, which were elevated by HFD. Analysis of the effect of HSE on paraoxonase 1, an antioxidant liver enzyme, revealed that HSE potentially regulates lipid peroxides and protects organs from oxidation-associated damage. The markers of liver damage such as serum alanine aminotransferase and aspartate aminotransferase levels that were elevated by HFD were also reduced on HSE treatment. The effects of HSE were as effective as treatment with anthocyanin; therefore the anthocyanins present in the HSE may play a crucial role in the protection established against HFD-induced obesity.

**Conclusions:**

In conclusion HSE administration constitutes an effective and viable treatment strategy against the development and consequences of obesity.

Obesity is a chronic metabolic disorder caused by imbalance in energy intake and energy expenditure. Obesity is associated with an increase in adipogenesis, the process whereby undifferentiated preadipocytes are converted to differentiated adipocytes (1). Various metabolic syndromes such as nonalcoholic fatty liver disease (NAFLD), insulin resistance, and hypertension are known to be associated with obesity (2, 3). Obesity is most commonly accompanied by liver damage, as dietary obesity promotes liver inflammation and disease (4). Because diets containing high-fat foods are becoming common, it is necessary to find suitable alternatives, such as phytochemicals, to ameliorate the effects of a high fat diet (HFD), thereby reducing the risk associated with obesity (5, 6).

Phytochemicals are known to possess antioxidant properties with effective anti-inflammatory, antiallergic, hepatoprotective, antithrombotic, antiviral, and anticarcinogenic activities (7–9). Among them, polyphenols such as resveratrol, catechins, and anthocyanins are of great interest due to their wide distribution in plants and potent health-promoting activities (10–12). Treatments with phytochemicals obtained from various sources have been demonstrated to regulate the levels of total cholesterol (TC), triglycerides (TG), high-density lipoprotein cholesterol (HDL-C) and low-density lipoprotein cholesterol (LDL-C) in HFD-fed rats (13–15).

*Hibiscus sabdariffa* L. (Malvaceae; common name “roselle”) is an attractive plant cultivated in Sudan and in Eastern Taiwan. *H. sabdariffa* is consumed as traditional Chinese rose tea and is used effectively against hypertension. Extracts from *H. sabdariffa* are known to possess effective antioxidant, antihyperlipidemic, antiatherosclerotic, and hepatoprotective properties. In our previous work, *H. sabdariffa* extract (HSE) was found to exhibit chemopreventive properties through mitogen-activated protein kinase (MAPK) signaling pathways (16). HSE has also been used in traditional medicines for its pharmacologic properties, which include hepatoprotection and antioxidant activities and provide protection against atherosclerosis and fatty liver disease (17–20).

Anthocyanins are widely known to be effective in controlling body weight gain, which is vital in the treatment and prevention of obesity (21). The floral extracts of *H. sabdariffa* are rich in anthocyanin contents, which have high therapeutic potential. Anthocyanins from blackberries (*Rubus* sp.), blueberries (*Vaccinium angustifolium*), mulberries (*Morus australis* Poir), and blood oranges (*Citrus sinensis* [L.] Osbeck) were shown to prevent obesity in mice fed HFD (22–26).

In this study, the anti-obesity activity of HSE was tested against HFD-induced obesity in Mesocricetus auratus hamster. HSE was found to be effective in controlling body weight gain and adipose deposition. Further HSE administration ameliorated lipid accumulation, lipid peroxide activities, and tissue damage in the livers of HFD-fed hamsters. The effect of HSE on HFD-induced obesity was very similar to that of anthocyanin (cyanidin-3-*O*-β-glucoside) used as a positive control.

## Materials and methods

### Preparation of HSE

The HSE extract was prepared following methods reported previously (27). *H. sabdariffa* was purchased from a Taitung County farmers’ association, Taitung, Taiwan, after confirming the identification by comparing with a sample in the herbarium (catalog number TAI074435) of the National Taiwan University, Pintung, Taiwan. Briefly, 20 g of *H. sabdariffa* was washed with distilled water and air-dried. *H. sabdariffa* was resuspended in 100 mL of distilled water at 4°C overnight. Next, the supernatant was filtered through two layers of gauze to remove the debris and then lyophilized. The dried HSE was stored at −20°C; before use it was reconstituted with water. Characterization of the components of HSE by high performance liquid chromatography showed that it contains 1.39% flavonoids, 2.48% anthocyanins, and 1.67% polyphenols. HSE polyphenols are composed of protocatechuic acid (8.62%), catechin (9.86%), epigallocatechin (10.11%), epigallocatechin gallate (20.34%), and caffeic acid (18.24%). The anthocyanins are composed of delphinidin (79%) and cyanidin (18%) (28).

### Animals and experimental design

All animal experimental procedures followed the protocols approved by the Institutional Animal Care and Use Committee of Chung Shan Medical University (CSMU IUCAC No. 513) in Taichung, Taiwan. Male hamsters, aged 8 weeks and weighing 120 g, were purchased from the National Laboratory Animal Breeding and Research Center (Taipei, Taiwan) and housed in standard laboratory conditions (18–23°C, 55–60% humidity, and 12 h light/dark cycle) for the first 1 week of adaptation to the environment and during the experiments that followed. All hamsters were randomly divided into six groups (*n*=8 per group) as follows: control (standard laboratory diet, PMI Nutrition International, Brentwood, MO, USA); HFD (standard laboratory diet containing 10% coconut oil and 0.5% cholesterol); HFD supplemented with 25, 50, or 100 mg of HSE per day; or HFD supplemented with anthocyanin (cyanidin-3-*O*-β-glucoside, Polyphenol Laboratories AS, Sandnes, Norway). After 10 weeks of feeding on these diets, the hamsters fasted for 12–14 h and were then euthanized; all of their blood as well as their livers were collected.

### Blood sample analysis

Blood samples were collected from the hamsters with EDTA tubes and immediately centrifuged at 1,500*g* for 10 min. The serum was then decanted and stored at 4°C. Biochemical examinations were performed within 1 h of specimen collection. Serum levels of TG, HDL-C, LDL-C, alanine transaminase (ALT), and aspartate aminotransferase (AST) were measured by using clinical chemistry reagent kits (Randox Laboratories, Antrim, UK). Serum paraoxonase 1 (PON-1) activity measurement was using EnzChek^®^ Paraoxonase Assay Kit (Life Technologies CO., Ltd. Taipei, Taiwan). The protocol was used basically according to the manual of the kit. First 10 µl of each serum sample was added into 50 µl of reaction buffer in a plate prewarmed to 37°C. Then 50 µl of paraoxonase substrate working solution was added and the mixture was transferred to a fluorescence microplate reader set to 37°C. The plate was read using excitation at 360 nm and emission at 450 nm immediately. The collection data was calibrated and analyzed using the standard curve assay suggested by the kit manual.

### Statistical analysis

All experiments were performed in triplicate. Significant differences were assessed using one-way ANOVA, and *p*<0.05 was considered statistically significant. The data are expressed as the mean±SEM.

## Results

### HSE protects hamsters from HFD-induced physiological changes

Feeding of HFD caused significant characteristic changes associated with obesity in hamsters within 10 weeks. The hamsters fed with HFD showed an increase in body weight and in the weight of gonadal fat tissue after administration (Table 1). The HSE treatment showed high efficiency in regulating the total body weight and the increased fat content. The anthocyanin treatment group showed a similar trend to that of the HSE group.

**Table 1:** Effects of HSE and anthocyanin supplement on obese hamsters

		High fat diet				
		
	Control	HSE (0 mg)	HSE (25 mg)	HSE (50 mg)	HSE (100 mg)	Anthocyanin (25 mg)
Body weight before administration	18.03±0.11	18.00±0.10	18.05±0.13	18.01±0.14	18.06±0.14	18.01±0.07
Body weight after administration	22.32±2.28	31.61±9.49#	27.68±8.46*	22.79±9.22*	20.9±9.52*	21.79±10.23*
Weight gain after administration	4.29±2.36	13.61±9.37	3.63±7.65	4.78±8.83	2.84±9.11	3.78±10.16
Gonadal fat tissue weight after administration	0.20±0.02	0.29±0.05#	0.24±0.04	0.22±0.05*	0.21±0.05*	0.22±0.06*

HSE, *Hibiscus sabdariffa* water extract; HFD, high-fat diet. Each value is expressed as the mean±SD (*n*=10 per group). Results were statistically analyzed with one-way ANOVA.

# *p*<0.05 compared with the control group.

* *p*<0.05 compared with the HFD group.

### HSE ameliorates the changes in serum markers induced by HFD

Obesity-associated serum markers such as the ratio of LDL-C to that of HDL-C were found to be significantly modulated in the hamster groups fed with HFD (Table 2). The treatment of HSE again proved to be very efficient in reducing the elevated serum constituents. Similarly the anthocyanin-treated groups also showed regulated levels of serum obesity markers.

**Table 2:** Effects of HSE and anthocyanin supplement on plasma lipid parameters levels in hamsters

		High fat diet				
		
	Control	HSE (0 g)	HSE (25 mg)	HSE (50 mg)	HSE (100 mg)	Anthocyanin (25 mg)
LDL-C (mg/dL)	75±9	311±53###	126±9***	105±8***	99±7*	113±9*
HDL-C (mg/dL)	38±4	70±7###	53±5***	44±6***	46±4*	50±2*
LDL-C/HDL-C ratio	1.97±0.23	4.44±0.75###	2.38±0.17*	2.38±0.18***	2.15±0.15***	2.26±0.18***

HDL-C, high-density lipoprotein cholesterol; LDL-C, low-density lipoprotein cholesterol. Each value is expressed as the mean±SD (*n*=10 per group). Results were statistically analyzed with one-way ANOVA. ^##^ *p*<0.05

### *p*<0.001 was compared with the control group.

* *p*<0.05

*** *p*<0.001 was compared with the HFD group.

### HSE reduces fat accumulation in hamster livers

**Fig. 1:**
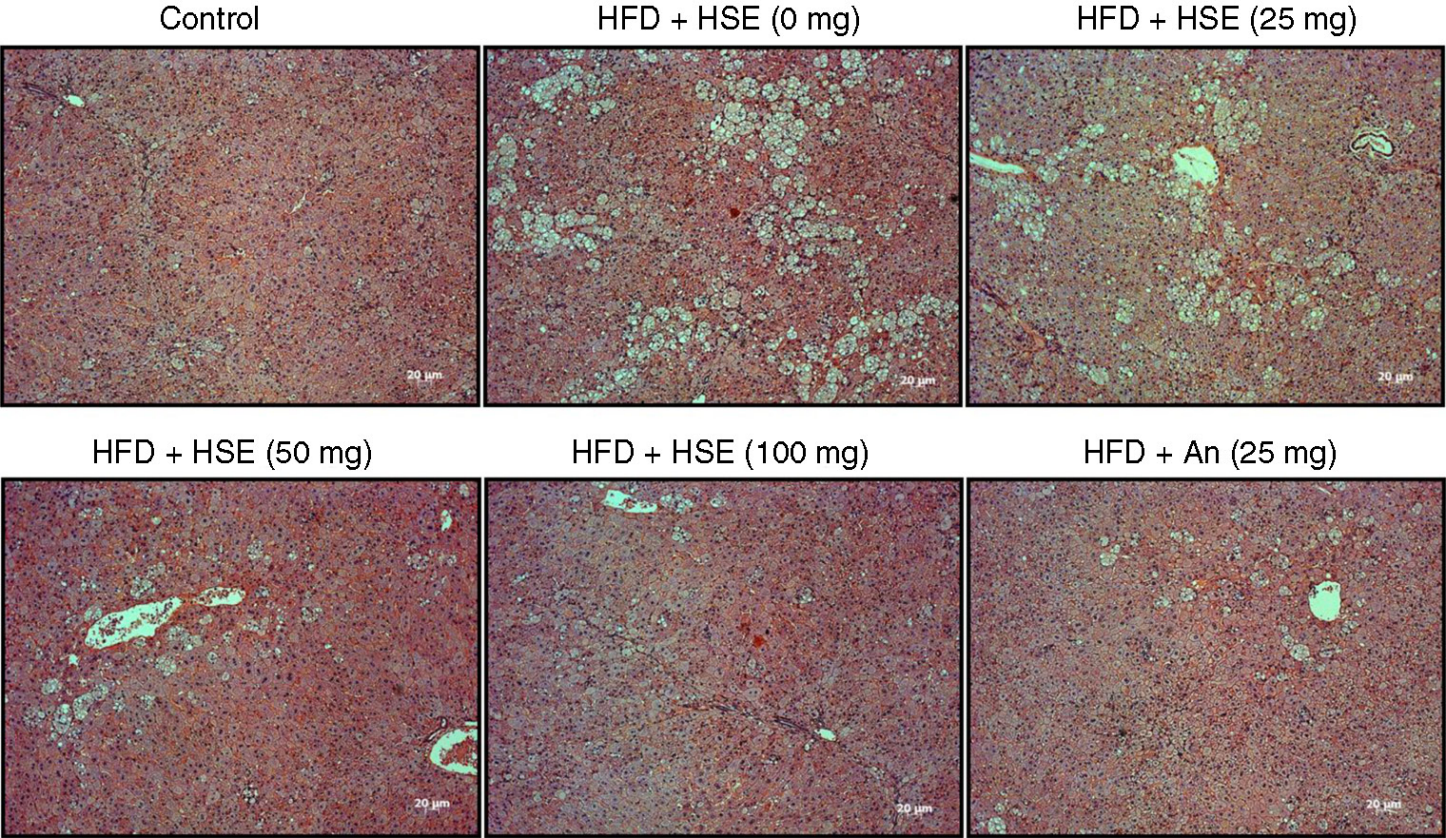
Effects of *Hibiscus sabdariffa* water extract (HSE) supplement on body weight and adipose tissue in HFD-fed hamsters. H and E staining of liver sections shows various degrees of fat accumulation in hamsters fed a normal diet (control) and hamsters fed with HFD along with different amounts of HSE or 25 mg anthocyanin.

The H and E staining of the hamster liver sections show extensive accumulation of fat in the livers of hamsters fed with HFD. However, the hamsters fed with 25, 50, or 100 mg of HSE showed a dose-dependent decrease in the amount of liver fat bodies. Treatment with 100 mg of HSE exhibited a high potential for ameliorating the fatty liver conditions caused by HFD (Fig. 1). A comparative analysis of the effect of HSE and that of anthocyanin on HFD-fed hamsters shows 100 mg of HSE treatment to be superior.

### HSE reduces HFD feeding-related modulations in hamster livers

**Fig. 2:**
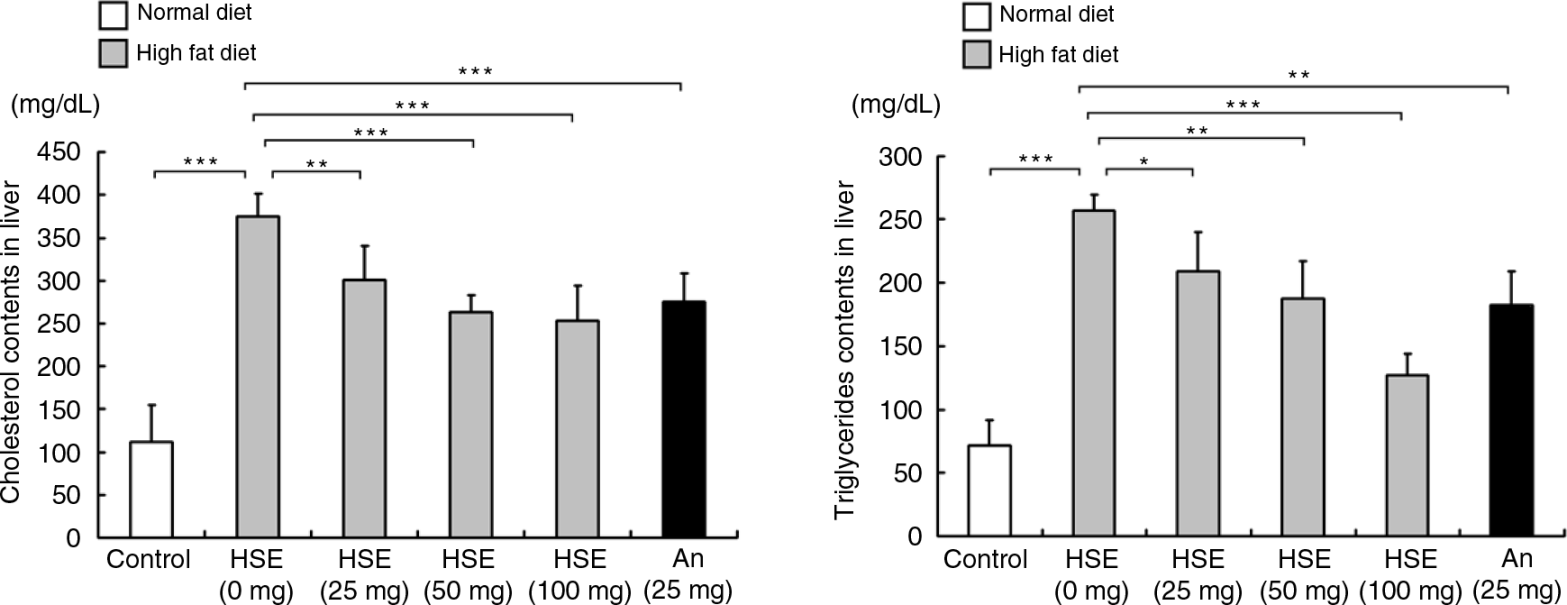
Effects of HSE supplement on serum lipid levels in HFD-fed hamsters. Serum cholesterol and triglyceride levels in hamsters fed a normal diet (control) and hamsters fed HFD along with different amounts of HSE or 25 mg anthocyanin. Data are shown as the mean±SD: *p<0.05, **p<0.01, ***p<0.001 compared with the HFD group.

HFD increased the liver cholesterol and TG levels significantly but the levels were decreased in the hamster groups treated with HSE (Fig. 2). The effect of HSE was dose-dependent and the 100 mg HSE treatment group showed better results than the anthocyanin-treated group.

### HSE potentially attenuates HFD induced atherosclerosis condition

**Fig. 3:**
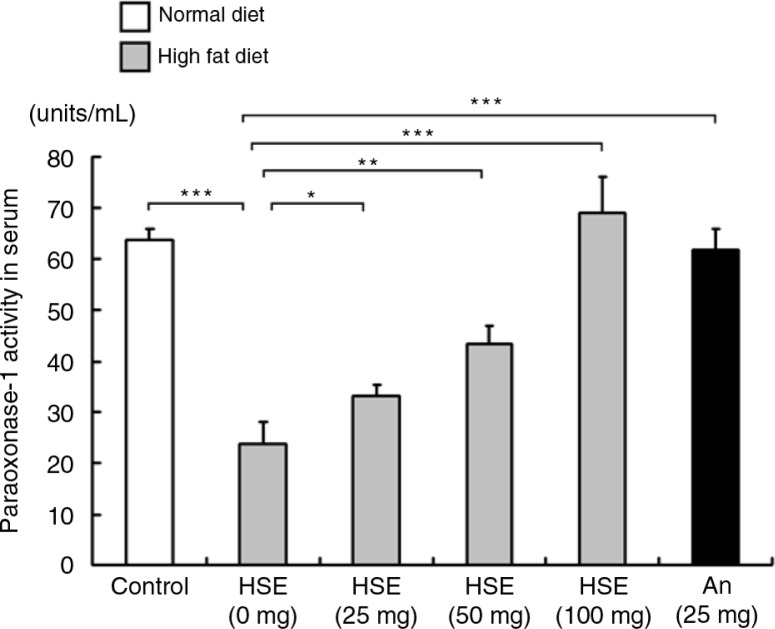
Effects of HSE supplement on paraoxonase 1 (PON-1) in HFD-fed hamsters. Serum PON-1 levels in hamsters fed a normal diet (control) and hamsters fed HFD along with different amounts of HSE or 25 mg anthocyanin. Data are shown as the mean±SD: *p<0.05, **p<0.01, ***p<0.001 compared with the HFD group.

The serum activity levels of PON-1, which is known for its anti-atherosclerotic property, decreased in the HFD-fed hamster groups. However, treatment with various concentrations of HSE increased the PON-1 activity levels in the hamster serum (Fig. 3). Therefore HSE treatment reduced the risk of atherosclerosis associated with HFD feeding.

### HSE regulates the ALT and AST level analysis

**Fig. 4:**
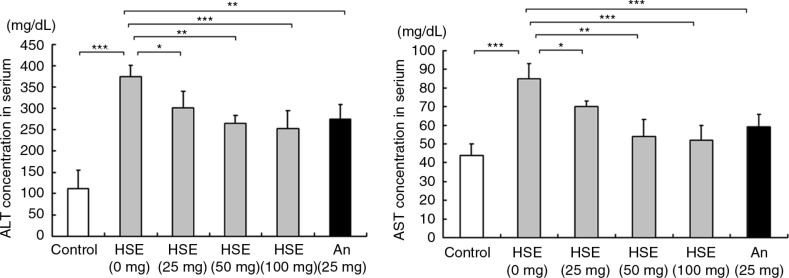
Effects of HSE supplement on serum alanine aminotransferase and aspartate aminotransferase in HFD-fed hamsters. Serum alanine aminotransferase and aspartate aminotransferase levels in hamsters fed a normal diet (control) and hamsters fed HFD along with different amounts of HSE or 25 mg anthocyanin. Data are shown as the mean±SD: *p<0.05, **p<0.01, ***p<0.001 compared with the HFD group.

Analysis of the serum ALT and AST levels show that ALT and AST (Fig. 4) levels increased in hamsters fed with HFD. The ALT and AST levels were, however, reduced in the hamsters treated with various doses of HSE and the results were similar to the effects of anthocyanin.

## Discussion

Obesity is considered to be a major risk factor associated with various diseases, including coronary heart disease, hypertension, type 2 diabetes mellitus, cancer, respiratory complications, and osteoarthritis (29). Recent reports have outlined that administration of an efficient anti-obesity drug may decrease energy/food intake and increase energy expenditure, decrease preadipocyte differentiation and proliferation, decrease lipogenesis, and increase lipolysis and fat oxidation (30). In this study, the anti-obesity effects of HSE were determined through body weight, fat mass, serum lipid profile, and biochemical examination of liver function in HFD-fed hamsters.

Our data revealed that both HSE and anthocyanin decrease the levels of serum and hepatic lipids such as TC and TG in HFD-fed hamsters, which could be attributed to the inhibition of lipid absorption in the gut. Dietary lipids are absorbed into the bloodstream and are then digested to be transported and stored in the liver and adipose tissues in the form of TG (16).

Blood samples of the HFD-fed hamsters showed an increase in the levels of TG; the ratio of LDL-C to HDL-C increased in the hamsters. The increase in cholesterol may potentially enhance the risk of fatty liver and atherosclerosis (31, 32). Oxidized LDL has been shown to be atherogenic and inhibition of the LDL oxidation by potent dietary antioxidants effectively attenuates atherosclerosis (33, 34). HSE is rich in high antioxidant components such as anthocyanins that can decrease the oxysterols in bile acid metabolism block the absorption of lipids into the liver by the bile salt system (35, 36). Protection of lipids from oxidation can be also achieved by serum PON-1, an HDL-associated esterase that can hydrolyze and reduce specific lipid peroxides in arterial cells and in lipoproteins in coronary and carotid lesions (37). Our results further show that HSE treatment effectively enhances the serum PON-1 levels, which were found to be lower in the HFD-fed hamsters. Therefore, HSE treatment can effectively decrease the adverse effects of serum lipid peroxides in the arteries.

Obesity is known to cause inflammation of the liver (38). To have a better understanding of the HFD effects on liver function, the enzyme activities of ALT and AST were measured (39). Our results showed that HFD indeed increased serum ALT and AST levels, revealing that HFD induced liver damage in hamsters fed with HFD (40). Both ALT and AST are leakage enzymes, and their elevation in circulation indicates significant hepatocellular damage. Toxicity, inflammation, hypoxia, and tissue trauma may form the underlying reason for their elevation (41, 42). Our results revealed that HSE treatment significantly reduced ALT and AST; therefore HSE ameliorated the potential liver damage caused by HFD. The reduction of liver enzymes after HSE treatment indicated a decrease in fat deposition and necrosis in liver cells (43).

Our previous work revealed that HSE efficiently reduced serum cholesterol levels in 42 volunteers after 4 weeks of administration (20). HFD has been known to increase the liver mitochondrial reactive oxygen species (ROS) production (44). ROS causes cell damage via the mechanism involving lipid peroxidation that leads to tissue injury, especially in the liver (45). Dietary polyphenols contain a number of phenolic hydroxyl groups and have demonstrated various beneficial effects, which is mainly due to their ROS scavenging activity (46). Polyphenolics are widely distributed in vegetables, fruits, and beverages and are present as an integral part of typical human diets (47, 48). Various polyphenol-rich sources such as tea, pomegranate, grape juice, and apples have demonstrated hepatic protection against various challenges (46, 48–51). Various herbal extracts, such as *Chrysanthemum morifolium* extract, *Morinda citrifolia* L. extract, and *Coix lacryma-jobi* L. extract, that are abundant in polyphenols have also been shown to provide efficient liver protection against obesity-related liver damage (52–55).

## Conclusions

To sum up, HSE treatment improved the HFD-induced obesity and lipid accumulation-induced damage in the liver in the animal model. Polyphenolic- and flavonoid-rich HSE treatment is also a safe therapy for HFD-induced obesity.
